# Advances and Trends in Omics Technology Development

**DOI:** 10.3389/fmed.2022.911861

**Published:** 2022-07-01

**Authors:** Xiaofeng Dai, Li Shen

**Affiliations:** Wuxi School of Medicine, Jiangnan University, Wuxi, China

**Keywords:** omics, next generation sequencing, third generation sequencing, mass spectrometry, redoxomics

## Abstract

The human history has witnessed the rapid development of technologies such as high-throughput sequencing and mass spectrometry that led to the concept of “omics” and methodological advancement in systematically interrogating a cellular system. Yet, the ever-growing types of molecules and regulatory mechanisms being discovered have been persistently transforming our understandings on the cellular machinery. This renders cell omics seemingly, like the universe, expand with no limit and our goal toward the complete harness of the cellular system merely impossible. Therefore, it is imperative to review what has been done and is being done to predict what can be done toward the translation of omics information to disease control with minimal cell perturbation. With a focus on the “four big omics,” i.e., genomics, transcriptomics, proteomics, metabolomics, we delineate hierarchies of these omics together with their epiomics and interactomics, and review technologies developed for interrogation. We predict, among others, redoxomics as an emerging omics layer that views cell decision toward the physiological or pathological state as a fine-tuned redox balance.

## Introduction

“OMICS,” defined as probing and analyzing large amount of data representing the structure and function of an entire makeup of a given biological system at a particular level, has substantially revolutionized our methodologies in interrogating biological systems. In other words, “top down” approaches, largely attributable to “omics” development, coupled with “bottom up” strategies to offer a holistic tool for efficient biological system investigation. The concept of dissecting complex disorders including cancers has been, accordingly, advanced from static delineation between cell malignant and heathy states in a low-throughput manner to spatio-temporal dynamic deconvolution of complex systems involving multi-layer modifications at genomic, transcriptomic, proteomic, and metabolic levels in a global-unbiased fashion.

Ever since the establishment of the first high-throughput technology, DNA microarray (1), technologies for omics exploration have been developed by leaps and bounds. Following the central dogma, omics technologies have been used to capture the static genomic alterations, temporal transcriptomic perturbations and alternative splicing, as well as spatio-temporal proteomic dynamics and post translational modifications (PTMs) (2). Beyond this, omics technologies have been expanded to analyze various omics at the epi-level (such as epigenome, epitranscriptome, epiproteome that are defined as the collection of all modifications of the referred omics beyond information it covered in a single cell), molecular interactions (i.e., varied levels of interactome), and disease associated hallmarks as metabolome and immunome. Multi-omics integration has become a prevailing trend for constructing a comprehensive causal relationship between molecular signatures and phenotypic manifestations of a particular disease, and single cell sequencing offers additional resolving power that enables investigations at a single cell level. This rapidly-developing and ever-growing field, omics, has empowered us to uncover the intricate molecular mechanism underlying different phenotypic manifestations of disordered traits in an overwhelming and systematic manner at a high accuracy. However, the complexity of the cellular behavior and its decision-making system may persistently drive the establishment of novel omics and associated techniques.

While we are running close to the truth in principle, the ever-growing knowledge on cellular omics persistently transforms our understandings toward cell machinery complexity that challenges our goal toward the fully harness of cell pathological state rewiring. It is, thus, time to comprehensively review what has been done and is being done in omics-relevant studies to forecast what can be done in “omics” as a shortcut toward our goal. Focusing on the four big omics, i.e., genomics, transcriptomics, proteomics and metabolics, their epiomics and pair-wise interactomics, this paper comprehensively reviews high-throughput technologies developed, and forecasts, among others, the emerging role of “redoxomics” on the cell machinery.

## Technology-Based Omics

**Figure 1:**
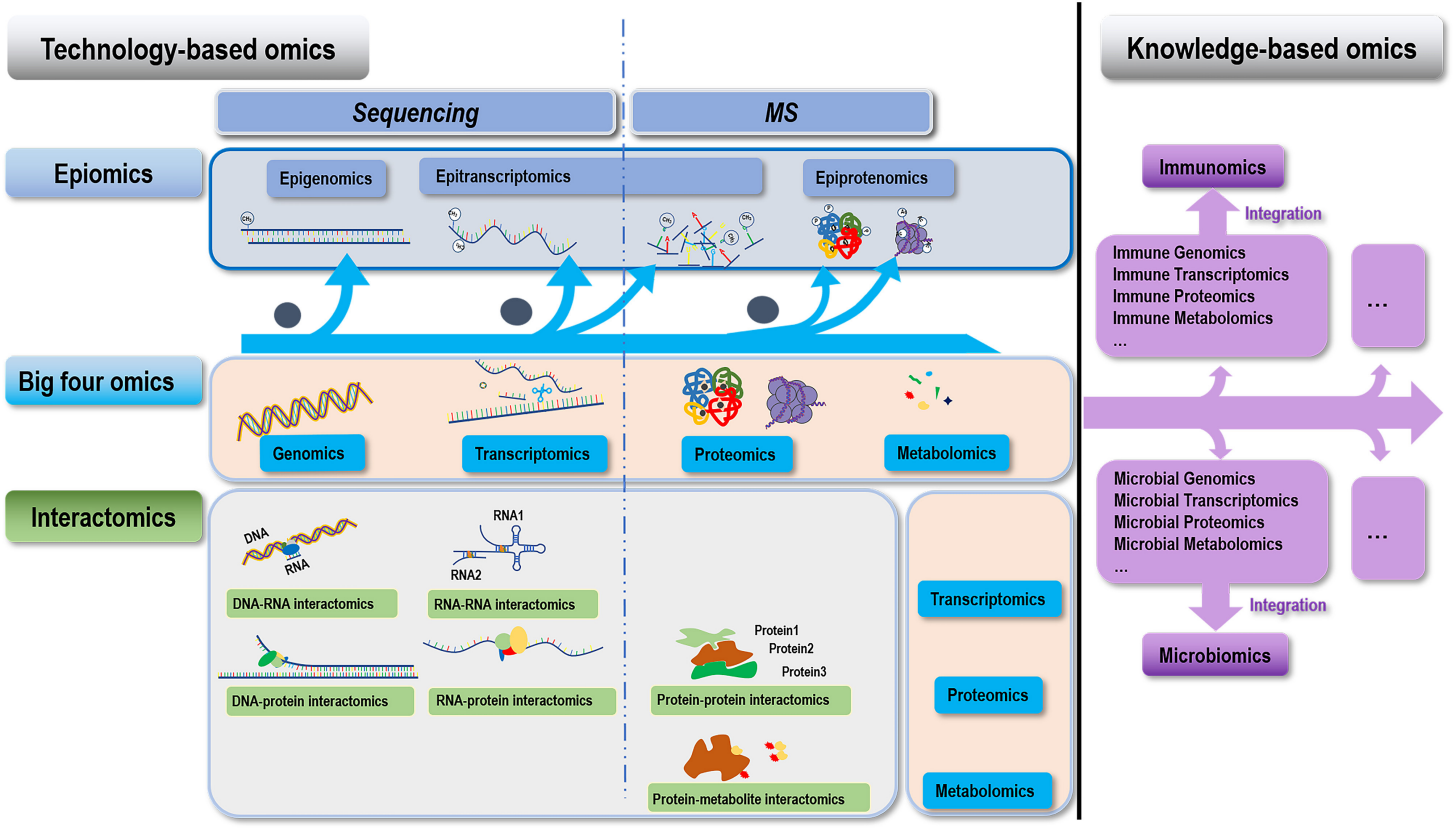
Conceptual illustration on the hierarchy of different omics covered in this paper. We classify omics technologies into two categories, i.e., technology- and knowledge- based. Technology-based omics are based on technologies developed for understanding the “central dogma,” which can be further divided into three groups, i.e., the “four big omics” (genomics, transcriptomics, proteomics, and metabolomics), epiomics (epigenomics, epitranscriptomics, and epiproteomics), and their interactomics (DNA-RNA interactomics, RNA-RNA interactomics, DNA-protein interactomics, RNA-protein interactomics, protein-protein interactomics, and protein-metabolite interactomics). Omics indicated by the horizontal (above) and vertical (right-hand side) pink boxes of each interactomic term constitute to its two interacting omics. Knowledge-based omics are developed to understand a particular knowledge domain in a systematic way through integrating multiple omics information. Examples of this category include immunomics, microbiomics, and beyond.

Sequencing and mass spectrometry (MS) are basic experimental tools availing in our tour in investigating the omics of a given biological system. While sequencing-based approaches are feasible for studies on genome, transcriptome, their epitomes and interactomes involving DNA/RNA, MS-based techniques can be used to interrogate proteome, metabolome, and interactomes that do not involve DNA/RNA (Figure 1, Table 1).

**Table 1:** Comparisons of high-throughput approaches for omics studies.

**Methods**		**Advantages**	**Disadvantages**	**References**
**Genomics**		
DNA microarray		• Inexpensive; • It allows focused detection of a defined number of targets, limiting information to only genes of interest; • Relatively mature.	• Inability to detect *de novo* transcripts; • Inaccurate when analyzing highly repetitive genomes; • The data are considered as being “noisy”; • Large sample size is required.	(3)
The first-generation sequencing	Sanger sequencing	• Long read lengths and high per-base accuracies.	• High cost and low throughput.	(4–6)
The next-generation sequencing	Cyclic-array sequencing	• Lower cost; • High throughput.	• The average single reading accuracy is low.	(3)
	Microelectrophoretic	• Low cost.	• Low throughput.	(3)
	Sequencing by hybridization	• Improved throughput by avoiding the electrophoresis step that allows more samples to be sequenced in parallel.	• A single sample must first be cloned, amplified and purified.	(4)
	Real-time observation of single molecules	• Higher speed and throughput; • Relatively low cost; • Easier gene library construction; • Higher level of parallelism.	• Short read lengths and less accuracy; • Cannot well capture some sequences.	(4, 5, 7)
The third-generation sequencing	PacBio	• Real long reads; • Extremely high accuracy; • Direct detection of epigenetic modifications; • No problem with repeats, low/high %GC.	• Expensive sequencer and relatively high cost per Gb; • Large amounts of starting material required for library; • Preparation; • High error rate at single pass; • Limited throughput per SMRT cell; • Maximum read length limited by polymerase processivity.	(8)
	ONT	• Real (ultra-) long reads that with no upper limit; • Cost-effective sequencers (MinION, GridION); • Direct detection of epigenetic modifications; • Extremely fast library preparation; • Direct sequencing of RNA and detection of RNA modifications.	• High overall error rate and systematic errors with homopolymers; • Large amount of starting materials required for library preparation; • Frequent changes of software versions, flow cells, and kits.	(8)
**Transcriptomics**				
RNA microarray		• Less expensive; • Technology is relatively mature.	• Inability to detect *de novo* transcripts; • Large sample size is required.	(6)
Tag-based methods	DGE seq	• More economical than traditional RNA sequencing for a given sequencing depth; • Provide a higher dynamic range of detection.	• Biases from fragmentation, adapter ligation and PCR can make tag-based data more prone to batch effects.	(9)
	3' end seq	• Direct sequencing of the 3' end.	• Generate a high frequency of truncated cDNA; • Sequence preference of RNA ligases can introduce bias.	(9)
Probe alternative splicing and gene fusion	SMRT	• Offers long reads.	• Costly, and has a high error-rate and low multiplexing capacity.	(9)
	SLR-RNA-Seq	• Delivers longer transcripts and more detected isoforms.	• Genome wide analysis is not possible; • Relatively high cost.	(9)
Targeted RNA sequencing	Target capture	• Greater complexity and uniformity; • Better uniformity.	More costly.	(9)
	Amplicon sequencing	• Low cost; • Higher on-target rates.	• Cannot involve complex analysis; • Lower uniformity.	(9)
Single cell RNA sequencing	CEL-seq2	• High sensitivities; • Detected more UMIs and genes per cell.	• Lower throughput.	(10)
	Drop-seq	• High throughput; • low cost.	• Lower sensitivities.	(10)
**Proteomics**				
High resolution MS methods	Orbitrap	• High resolving power; • Better resolution than FT-ICR at higher m/z; • Lower cost than FT-ICR; • The instrument is much smaller and requires less maintenance than FT-ICR.	• The only fragmentation method available is ion trap-based CID, a method that has limitations on modified peptides with important PTMs;•The practical accurate mass MS/MS scan rate is slow; • Very prone to space-charge effects.	(11)
	MALDI-TOF-TOF	• Fast scanning speed; • High throughput.	• Low resolving power.	(11)
	FT-ICR	• Very high mass accuracy and resolving power.	• Equipment takes up more space; • High cost; • low scan speeds.	(11)
Low resolution MS approaches	Quadrupole	• Low cost; • Compact shape and size; • Rugged and reliable for long periods of time.	• Less suitable for pulsed ion sources; • Suffer from both limited mass ranges and poor resolution.	(11)
	Ion-trap	• Improved sensitivity; • Compact shape and size.	• Low resolving power.	(11)
Tandem mass spectrometric techniques	CID	• Mature technology with wide applications.	• Cannot capture unstable PTM information.	(12)
	ECD	• The retention of labile groups is far superior than CID; • Capable of producing product ions that are complementary to those observed using CID.	• Negative ions formed by ESI are usually not amenable; • It has received broad commercial implementation only on FT-ICR MS.	(12, 13)
	ETD	• The retention of labile groups is far superior than CID; • Capable of producing product ions that are complementary to those observed using CID; • Can be used in combination with various mass spectrometers.	• Negative ions formed by ESI are usually not amenable; • The fragmentation efficiency is lower than ECD.	(12–14)
	EID	• Can be used to induce fragmentation in singly protonated or deprotonated ions.	• Negative ions formed by ESI are usually not amenable.	(12)
**Metabolomics**				
Spectroscopy	FT-IR spectroscopy	• Low cost; • Simple operation.	• A long preparation process may lead to errors.	(15, 16)
	Raman spectroscopy	• Non-destructive, non-invasive; • Minimal sample preparation; • Label free, no dyes and toxic waste products; • High specificity; • Simultaneous detection of macromolecules.	• Low sensitivity; • Weak Raman signals leads to long acquisition time; • Video rate imaging almost impossible due to low scattering efficiency and long measurement time; • Sophisticated data analysis is needed.	(16, 17)
	NMR spectroscopy	• Simple sample preparation and highly reproducible molecule quantification; • Nondestructive, nonbiased, requires little or no chromatographic separation or chemical derivatization.	• Less sensitive than LC/MS and GC/MS.	(18, 19)
MS	MS	• Mature technology with wide applications; • Good method of choice to identify and quantify complex protein samples.	• MS data are less reproducible than NMR spectroscopy; • The sample cannot be recovered; • *In vivo* fluxomic is not possible with MS, and isotope mapping is more difficult.	(19, 20)
	MS/MS	• It compensates for the poor chromatographic ability of LC/GC.	• Not all molecules can be efficiently fragmented or detected.	(21, 22)
	GC-MS	Mature technology and cheap price.	• Analytes have to be volatile or volatilizible by derivatization; • Cleaning of ion source requires venting of the system, involves a large number of parts; • Time consuming.	(21)
	LC-MS	• No limitations by molecular mass or polarity of target analytes; • Ion source can be cleaned without venting; • Relatively few parts need to be cleaned; • Short time consuming and partially automated.	• More cost; • The technology is not mature enough.	(21)
**Epigenomics**				
Hi-C		• High resolution; • High throughput; • Highly parallel.	• Cannot capture the fine detail of sub-nuclear compartments; • Cannot measure the dynamics of interactions between multiple genomic loci.	(23)
MiGS		• Can analyze whole genome methylation; • Has better specificity and sensitivity than conventional methods of DNA methylation analysis.	• The description of methylation is not a single base pair resolution.	(24)
**Epitranscriptomics**				
Enzyme-based *in vitro*	PARS	• Increased sensitivity by sequencing both single- and double-stranded regions.	• RNA was folded *in vitro*.	(25)
	FragSeq	• Simple and fast protocol; • Accompanied with modifiable software.	• Does not consider single-hit kinetics that may lead to RNA restructuring after cleavage.	(26, 27)
	PARTE	• Measures melting temperature; • Single-nucleotide resolution; • Preserves *in vivo* RNA modifications; • Can infer RNA regulatory motifs.	• Introns and lowly expressed antisense or cryptic unstable transcripts are not well-interrogated; • RNA structures that require co-transcriptional folding or native protein-RNA interactions may not be correctly preserved.	(25, 28)
Chemical-based *in vitro*	Mod-seq	• Can probe structures of long RNAs *in vivo*; • Single-nucleotide resolution.	• Limited to the analysis of two bases (As and Cs).	(25)
	Structure-seq	• Single-nucleotide resolution; • Applicable to both *in vitro* and *in vivo* analyses.	• Limited to the analysis of two bases (As and Cs); • RNA-binding proteins can block DMS activity.	(25)
	DMS-seq	• Identifies RNA structure in native conditions; • Single-nucleotide resolution.	• Limited to the analysis of two bases (As and Cs); • RNA-binding proteins can block DMS activity.	(25)
Chemical-based *in vivo*	CIRS-seq	• Single-nucleotide resolution; • Can identify structural requirements for RNA-binding proteins; • Can accurately predict secondary RNA structures, and reveal features of mRNAs and ncRNAs.	• Uses DMS to methylate the N1 of adenosine and N3 of cytosine residues, and uses CMC to modify pseudouridines, where DMS and CMS may react with non-secondary RNA structures.	(25)
	SHAPE-MaP	• Can be customized for different applications; • Applicable to analysis of long RNAs; • Can infer structural changes of single-nucleotide and other allelic polymorphisms.	• Length of the RNA must be at least ~150nt for the randomer and native workflow, and at least ~40nt for the small-RNA workflow.	(25)
	icSHAPE	• Measures base flexibility; • Single-nucleotide resolution.	• Limited to the analysis of relatively short (300nt) *in vitro* transcribed RNAs.	(25)
	MARIO	• Many-to-many mapping; • Incorporation of an adaptor between two RNA molecules increases ligation efficiency and improves accuracy in sequence mapping; • Reports both between- and within-molecule interactions.	• Loses RNA duplexes that are not associated with any proteins.	(25)
RIP-seq		• Mature technology; • High throughput.	• The washing conditions are quite strict; • RNAs bound to RBPs with low-affinity may not be recovered; • Kinetically unstably bound RBPs may dissociate from their RNA targets.	(29)
LAIC-seq		• Could differentiate m6A methylation levels between mRNA isoforms without prior fragmentation.	• Losing the positional information.	(29)
miCLIP-seq		• M6A is detected with high specificity and sensitivity; • Excellent spatial resolution.	• The method is dependent on m6A-specific antibodies, suffering from poor reproducibility and complicated process; • Due to the low cross-linking efficiency, the number of m6A sites recognized is limited.	(29)
m1A-MAP		• Reveal distinct classes of base-resolution m1A methylome in the nuclear- and mitochondrial-encoded transcripts.	• Large sample size is required.	(29)
m7G-MeRIP-seq		• Precisely map the m7G methylomes in RNA.	• The mild chemical reactions for selective m7G reduction and depurination could not achieve quantitative yields.	(29)
RNA BisSeq		• Can accurately identify m5C sites; • Mature technology.	• It may be disturbed by some cytosine modifications other than m5C; • Limited resolution and requirement on large amounts of starting material.	(29)
MAZTER-seq		• Allows detecting and quantifying m6A levels at endogenous sites; • Allows rapid readouts on m6A Levels at individual loci; • Allows quantitative evaluation of sites identified via miCLIP.	• Allows quantification of only a subset of m6A sites that both occur at ACA sites and are within suitable distances of adjacent ACA sites; • For absolute (but not relative) quantification, cleavage efficiencies need to be normalized by their counterparts in methylation deficient backgrounds; • Is not entirely exclusive to ACA sites.	(29)
m6A-REF-seq		• High throughput; • High reliability and accuracy; • Independent antibody; • Less sample size and time required.	• It can only identify ~16 to 25% m6A sites because of the restrictions of MazF that specifically recognizes the ACA motif.	(29)
LC-MS/MS		• The presence and quantification of all RNA modifications can be determined.	• Requires large amount of input samples; • Does not provide information on the location of the modified positions.	(30)
**Epiproteomics**				
Microsequencing		• Mature technology.	• Time-consuming and requires a large amount of highly purified sample.	(31)
Western blotting		• Simple operation; • Mature technology; • Low cost.	• It is an error-prone method due to its time-consuming multistep protocol; • It is difficult to detect low abundance proteins; • Analysis of multiple proteins from a single sample run is often cumbersome; • Detection of some large molecular weight proteins (>500 kDa) can be problematic.	(32, 33)
Immunofluorescence analysis		• Permits visualization of virtually many components in any given tissue or cell type; • A variety of sample conditions can be employed.	• Usually restricted to permeabilized cells or extracellular or endocytosed proteins.	(33–35)
ChIP		• High resolution; • Not affected by noise.	• Sequencing errors may occur at the end of each read; • Requirement on correct sample loading amount as too little sample leads to too few tages and too much sample results in florescent labels too close to one another; • High cost.	(36)
MS		• Enabled the characterization of protein PTMs in a high-throughput manner; • Enable unbiased profiling of diverse modifications simultaneously; • Enable quantitative analysis of protein modifications; • Enable *de novo* identification of unknown modification patterns.	• False positive identification will be introduced during data verification; • The dynamic range is not optimal yet.	(33, 37)
**DNA-RNA interactomics**				
Mapping genome-wide locations of a specific RNA	ChIRP	• Tilling the entire transcript with antisense DNA.	• Limited to analyzing RNA at a time.	(25)
	CHART	• Tilling the RNase H accessible region by antisense DNA.	• Limited to analyzing RNA at a time.	(25)
	RAP	Tilling the entire transcript with complimentary RNA.	• Limited to analyzing RNA at a time; • Limited to analysis of long RNA.	(25)
Mapping all chromatin-interacting RNAs together with their genomic interacting regions	MARGI	• Many-to-many mapping; • Captures interaction at native conditions.	• Requires a large number (10^7^) of cells.	(25)
	ChAR-seq	• Many-to-many mapping; • Proximity ligation is performed in nuclei, which reduces nonspecific interactions.	• Only sequencing reads that cover the entire bridge sequence are informative, reducing the number of informative reads.	(25)
	GRID-seq	• Many-to-many mapping; • Proximity ligation is performed in nuclei, which reduces nonspecific interactions.	• The informative sequence lengths on the RNA side and the DNA side are both limited to 20 bases, resulting in challenges in unambiguous sequence mapping.	(25)
**RNA-RNA interactomics**				
hiCLIP		• Incorporation of an adaptor between two RNA molecules increases ligation efficiency and improves accuracy in sequence mapping.	• Requires prior knowledge of an RNA-binding protein; • Requires a good antibody; • No *in vivo* crosslinking step may incur challenges in differentiating bona fide and spurious RNA attachments.	(25)
PARIS		• Many-to-many mapping.	• 4'-Aminomethyl trioxsalen (AMT) preferentially crosslinks pyrimidine bases and may introduce bias.	(25)
SPLASH		• Improves signal-to-noise ratio by leveraging biotinylated psoralen; • Many-to-many mapping.	• Psoralen preferentially crosslinks pyrimidine bases and may introduce bias.	(25)
LIGR-seq		• Many-to-many mapping.	• AMT preferentially crosslinks pyrimidine bases and may introduce bias.	(25)
**DNA-Protein interactomics**				
ACE		• It can be a technique of choice to validate high throughput screening results; • Ease to use in both execution and data evaluation; • Mass application Tags; • Availability of models and software.	• The higher the analyte concentration, the bigger the systematic error will be.	(38)
ChIP-Chip		• technology with wide applications.	• Lots of cells are generally needed to obtain a robust result.	(38)
SELEX		• Mature technology with wide applications; • Strong ability to select aptamers.	• Prior exhaustive knowledge of protein target and high purity recombinant protein is necessary prior to selection of aptamers.	(38)
**RNA-Protein interactomics**				
CLIP-seq		• Cross linking occurs between RNA and protein before cell death.	• Large sample size is required.	(39–41)
CLASH		Stringent purification conditions remove nonphysiological interactions.	• Requires prior knowledge of an RNA-binding protein; • Requires a good antibody.	(25)
**Protein-Protein interactomics**				
Y2H		• Mature technology.	• Cannot identify multi-protein complexes in one run.	(41, 42)
LC-MS/MS		• Can tag several members of a complex; • Can detect real complexes in physiological settings.	• May miss some complexes that are not present under the given conditions; • Tagging may disturb complex formation; • Loosely associated components may be washed off during purification.	(41, 43)
coIP-MS		• Can rapidly identify multiple interacting proteins; • Applicable to different cell lines and species.	• The outcome is dependent on the efficiency of the antibody immunoprecipitating the bait protein.	(41, 44)
AlphaLISA		• Can study a wide range of analytes; • Can detect interactions with a wide range of affinities; • Easy to use.	• Excess target protein may oversaturate the donor or acceptor beads that results in a progressive signal decrease.	(45)
**Protein-metabolite interactomics**				
Protein tagging		• Can identify interacting metabolites for a specific protein.	• Low throughput.	(46)
Metabolite modification		• Available for a wide range of compound classes.	• Limited to compounds chemically stable over the course of the experiment.	(46)
PROMIS		• Low false positives related to a high concentration of the bait molecule; • Low false negatives related to small-molecule modifications.	• Poorly predictive.	(47)
NMR-based approach		• Widely applicable; • Can simultaneously detect the impact of several metabolites.	• Does not directly translate into changes in protein activity due to restrictions to protein-metabolite binding; • Requries moderate sample size; • The minimum size of the protein target should be > 10–30 kDa.	(48)
NMR relaxometry		• No separation step during sample preparation; • Can probe weak transient interactions; • The analysis is quantitative.	• Less sensitive than a state-of-the-art NMR system.	(49)

### Sequencing-Based Omics

#### Genomics

Genomic techniques are dedicated to investigate the inter-individual variations at both the germline and somatic levels via sequencing the genome of interest. The development from DNA microarray technology (50), first generation Sanger sequencing (51), second generation massively parallel sequencing, also known as the next generation sequencing (NGS) (52), and the eventual third generation of long reads sequencing (TGS) (53) have enabled the sequencing of the whole genome/exome with sufficient in-depth to characterize the mutational landscape of a given sample.

The DNA microarray technology was firstly established by Schena et al. (1), where thousands of probes were fixed to a surface and samples were labeled with fluorescent dyes for detection after hybridization (54). There are two types of DNA microarrays, i.e., 2-channel and 1-channel arrays, with Agilent (55) and Affymetrix GeneChip (56) being the typical 2- and 1-channel commercial array, respectively. In a 2-channel array, the array slides are fabricated by spotting with cDNA fragments or oligonucleotide probes; after hybridizing both samples, labeled by two types of fluorescent dyes such as Cy®5 and Cy®3, on the array, the gene expression of treated sample relative to the control is quantified by the ratio of the 2-channel intensities of each spot (57). In a 1-channel array, the oligonucleotide probes are synthesized on the slide surface to hybridize the fluorescence-labeled sample cDNAs, where the absolute intensity of hybridization signal is measured (58). As a variation of 1-channel array, Illumina BeadArray synthesizes barcoded probes on the surface of microbeads (59) (https://www.ncbi.nlm.nih.gov/probe/docs/techbeadarray). The DNA microarray technology is relatively mature, with various well-established experimental platforms and analytical tools available (60). Yet, the main drawback of DNA microarray technologies lies in its inability to detect *de novo* transcripts, since such technologies rely on probes designed according to known nucleotide sequences. Besides, DNA microarray is not a feasible platform when analyzing highly repetitive genomes due to the high occurrence of cross-hybridization events that may lead to inaccurate signal intensity estimation (61).

Sanger sequencing, also known as the first generation of DNA sequencing, was invented in 1977 (62). It is based on the selective incorporation of chain-terminating dideoxynucleotides by DNA polymerase during *in vitro* DNA replication. With the relatively long read length (i.e., up to ~1,000 bp) and high per-base accuracy (i.e., ~99.999%) (7), Sanger sequencing has been used to achieve a number of monumental accomplishments such as the completion of the Human Genome Project (63), and dominated this filed for almost 30 years (62, 63). Yet, it suffers from high cost and low throughput that calls for novel technologies delivering fast, inexpensive, and accurate solutions (62, 64).

NGS genome sequencing, comprised of primarily four categories, i.e., cyclic-array sequencing (65, 66), microelectrophoretic methods (67), sequencing by hybridization (68), and real-time observation of single molecules (69, 70), has dramatically improved the speed and scalability of genome sequencing. Taking cyclic-array sequencing as an example, the throughput has been substantially improved taking advantages of iterative cycles of enzymatic catalytic processes (4). Several commercial products are of this kind such as 454 Genome Sequencers (Roche Life Science, USA) (66), Illumina Genome Analyzer (Illumina, USA) (71), and SOLiD platform (Applied Biosystems, USA) (72), which have made milestone contributions to the omics field. However, Roche454 Genome Sequencers and the SOLiD platform quitted the market later due to, e.g., poor market acceptance, leaving Illumina the sole company dominating this field. Many mainstream products are from Illumina including, e.g., the MiSeq series such as MiSeq FGx, HiSeq series such as HiSeq X10, NextSeq series such as NextSeq550, and NovaSeq series such as NovaSeq6000. Beijing Genomics Institute (BGI), after the acquisition of Complete Genomics (CG), has entered the sequencing market and become an emerging institution capable of sequencer development, with BGIseq500 and NDBseq-T7 being its representative products. Other NGS platforms such as Ion Torrent (Thermo Fisher) also take market shares. NGS outweighs Sanger sequencing in higher speed and throughput (e.g., >106 reads/array in cyclic array sequencing), easier gene library construction, higher level of parallelism, and less costly in clinical practice [i.e., saving 30–1,249$/patient for cancer diagnosis (73)]. However, NGS suffers from the short read lengths it generated (averaged read length ranges from 32 to 330 bp) that leads to at least 10 folds less accuracy than Sanger sequencing (62, 64). Importantly, these short-read methods cannot well capture structural variants (SVs), repetitive elements, high/low GC content, or sequences with multiple homologous elements in the genome (74, 75). Also, the call for lowering down the overall cost persists as it still costs 1–60$/megabase despite the fact that the cost has already been lowered-down by several orders of magnitude as compared with Sanger sequencing.

TGS, the third revolution in sequencing technology as enabled by Pacific Biosciences (PacBio, 2011) (76) and Oxford Nanopore Technologies (ONT, 2014) (77), is a single molecular and real-time sequencing technique that allows for the long-read sequencing with low alignment and mapping errors during library construction. PacBio adopts the single molecule real-time (SMRT) technique, where ssDNA templates replicate during DNA library preparation automatically. PacBio SMRT has two sequencing modes, i.e., circular consensus sequencing (CCS) and continuous long read (CLR) sequencing, which differ in read length and error rate. While CCS has a higher accuracy at the sacrifice of read length by adopting a circular ssDNA template, CLR outweighs in getting higher coverage of ultra-long insert molecules that can substantially improve the assembly quality. During PacBio SMRT sequencing, the fluorescence signals are activated by a laser during the incorporation of a labeled dNTPs into DNA, and the color and duration of the emitted signals are recorded in real time during cell flow that is equipped with zero mode waveguides (78). In the ONT system, nanopores are inserted in an electrical resistant membrane, where a potential is applied across the membrane to enable a current flow through the nanopore, and signals are measured as characteristic disruptions in the current for each specific single molecule. A hairpin structure is designed to ligate the double DNA strands (dsDNA) during DNA library construction to enable the system read both DNA strands in one continuous read. The dsDNA is attached to the pore by the bound polymerase or helicase enzyme, and the signal of each nucleotide is captured as a characteristic disruption in the electrical current during sequencing while dsDNA moves through the nanopore (79). ONT can detect hundreds of kilobases in one continuous read, and sequence ultra-long reads (ULRs), i.e., with the length over 300 kb or even up to 1 million bp (80). Besides, some ONT sequencers are in the pocket-size that are portable without sophisticated laboratory setup, offering additional flexibility (81).

It is worth mentioning that, despite the rapid development and increasing popularity of NGS and TGS, the DNA microarray technology still gains favor in genome-wide association studies (GWAS) for the sake of economy, and there is a gaining momentum to combine DNA microarray and NGS/TGS in genotyping toward increased resolution of population-specific haplotypes and imputation strength (82).

Genomic sequencing technologies have been applied to characterize many genetic disorders (such as highly identical segmental duplications that account for over 5% of the human genome and are enriched in the short arm of the chromosome 16 (83) and diseases associated with BRCA1/2 mutations (84)), identify intratypic sequence variations [such as that of SARS-CoV-2 variants (85) and bovine papillomaviruses (86)], interrogate the genomic landscape of complex diseases [such as endometrial cancers (87) and thyroid carcinomas (88)], and discover novel alleles of polymorphic gene clusters in the human genome [such as that of the HLA system (89, 90)].

#### Transcriptomics

Unlike genome, transcriptome is dynamic and composed of diversified players. It is subjected to alterations imposed by cell development stage, internal and/or external stimuli, and the time point at which the signals are measured. Traditional transcriptome refers to mRNA transcripts, but can also be generalized to include other types of transcripts such as microRNAs (miRNAs), long non-coding RNAs (lncRNAs), and circular RNAs (circRNA). Transcriptomics techniques aim to detect and quantify RNA molecules transcribed from a particular genome at a given time (91).

Prior to the advent of NGS, RNA microarray was used as the conventional experimental technique to detect mRNA alterations within cells of interest at different stages in a high-throughput manner. RNA microarrays can be used to profile differentially expressed genes and identify markers capable of distinguishing cells between the normal and cancer states by concomitantly quantifying the relative mRNA abundance of thousands of genes.

Leveraged by the establishment of NGS technologies, RNA sequencing becomes possible that can be used to identify the presence and abundance of RNA transcripts in an unbiased and high throughput manner. Similar to DNA sequencing, *de novo* transcripts can be identified using RNA sequencing techniques given its independence on existing probes. Aided with the RNA sequencing technology, a vast amount and diversified types of non-coding RNAs (ncRNAs) have been discovered and found to be pervasively transcribed from the intergenic and intronic genome regions (92). This has substantially revolutionized our concept toward the complexity of mammalian transcriptome and the regulatory mechanisms leading to complex diseases such as cancers (93). RNA sequencing is commonly performed using DNA sequencing instruments given the platform compatibility (94) and technical maturity of commercially available DNA sequencing instruments (95), despite the possibility on direct RNA sequencing (96).

In addition to the whole transcriptome, a myriad of RNA sequencing platforms have been established to achieve *ad hoc* tasks. These techniques include tag-based methods (using one fragment to represent a transcript) such as digital gene expression (DEG) sequencing and 3' end sequencing, sequencing approaches to probe alternative splicing and gene fusion, targeted RNA sequencing, and single cell RNA sequencing (9). DEG sequencing is a deep sequencing approach derived from serial analysis of gene expression (SAGE) that is more economical than traditional RNA sequencing for a given sequencing depth (97–99). 3' end sequencing has been developed to interrogate alternative cleavage and poly(A) sites, which is comprised of approaches utilizing oligo (dT) for reverse transcription such as PAS sequencing (100), poly A sequencing (101), 3'T-fill (102), methods using RNA-based ligation to capture the 3' end fragments such as 3P sequencing (103) and 3'READS (104), and methods examining both the 3' end and the poly(A) tail length simultaneously such as TAIL sequencing (105) and PAT sequencing (106). Sequencing techniques such as RASL sequencing (107) can be used to analyze splice junction sites through the use of oligo pairs to target specific exon-exon junction sequences. Fusion events are typically revealed by reads containing fusion junctions or differences in expression between the 5' and 3' ends of genes that are fused. Several approaches have been established to specifically achieve this goal. As commercialized by NuGene and ArcherDX (108, 109), fusion genes are identified by amplicon-based sequencing through the use of two sequence-specific primers together with a common primer targeting the adapter sequence. Other approaches for fusion gene detection include RNA sequencing reads enrichment and exon capture (9). Sequencing a selected set of transcripts is sometimes desirable, leading to the development of targeted RNA sequencing. Approaches fell into this category include “target capture” (110–112) and “amplicon sequencing” (109, 113, 114). Driven by the demand of uncovering signals averaged out by examining the behavior of bulk cells at the population level, single cell sequencing has been evolved into a unique area for interrogating all levels of omics, where transcriptome is the first and most well-studied omics that has been interrogated at a single cell level. Sequencing techniques include, e.g., CEL-seq2 (115), STRT-seq (116), and Drop-seq (117).

Sequencing approaches have been established for interrogating other types of RNA species besides mRNA. Small non-coding RNAs such as miRNAs, piRNAs, and endosiRNAs are short in size, i.e., typically below 30 nt. Methods capable of separating them from contaminant DNAs are needed that include electrophoresis separation and blocking the 3′ adapter from ligating with the 5′ adapter by adding the reverse transcription primer (9). CircRNAs are generated by back-splicing (118), which can be sequenced by digesting and removing linear RNAs using exonuclease R, followed by regular RNA sequencing (9).

A growing number of platforms have been established to investigate RNAs at different stages of biogenesis, metabolism, and interactions with molecules such as proteins and other RNAs. These approaches share similar protocols in cDNA library preparation, but differ significantly in RNA-capturing that ranges from RNase protection to immunoprecipitation and to metabolic labeling (9).

Transcriptomic sequencing approaches have been widely used in medical studies, such as constructing the transcriptomic signatures of intestinal failure-associated liver diseases (119), revealing the pathogenesis of COVID-19 (120), identifying diagnostic biomarkers and therapeutic targets of multiple meylomas (121), and deciphering cell heterogeneity during osteogenesis of human adipose-derived mesenchymal stem cells (122).

#### Epigenomics

Epigenomics explains alterations in the regulation of gene activities that function without modifying genetic sequences, which serves as a major regulatory mechanism on gene transcription (123). It involves characterization of higher order chromatin structure (also constitutes to the DNA-DNA interactome) and DNA/RNA modifications such as DNA/RNA methylation (124).

Hi-C is a comprehensive technique developed to capture chromosome conformation, where chromatin is crosslinked with formaldehyde, digested, and re-ligated such that only DNA fragments covalently linked together are ligated. In Hi-C, a biotin-labeled nucleotide is incorporated at the ligation junction to enable selective purification of chimeric DNA ligation products followed by deep sequencing, where the ligation products contain the physical information of their genomic location origin and 3D genome organization (23).

Chemical modulations on certain DNA base as represented by DNA methylation may create dramatic impact on gene expression. Whole genome bisulfite sequencing represents a standard approach for methylated Cytosine base detection, which involves treating genomic DNA using sodium bisulfite followed by sequencing to generate a genome-wide landscape of methylated Cytosine at a single base resolution. MBD-isolated genome sequencing (MiGS) is another tool for whole genome methylation profiling that relies on the precipitation of methylated DNA by the recombinant methyl-CpG binding domain of MBD2 followed by sequencing (24).

Illumina short-read sequencing has been coupled with immunoprecipitation for DNA modification detection which, though being feasible for identifying epigenetic alterations in broad genomic regions, cannot reach the resolution at a single base level nor differentiate reads from different cells. Long-read sequencing technologies such as PacBio and Oxford nanopore sequencing techniques have thus been adapted for epigenome interrogation. PacBio SMRT, which monitors the polymerase in real time during DNA sequencing, detects epigenetic modifications by monitoring the inter-pulse durations in the reading rate of a polymerase as a result of kinetic variation (125). ONT for DNA modification detection relies on the different electric current alterations generated by molecules of different forms (e.g., methylated nucleotides) when they pass through a nanopore (126). Several groups have demonstrated the feasibility of TGS technologies in detecting DNA modifications, and tools such as Multi-MotifMaker (125) and NanoMod (126) have been designed accordingly for large scale *de novo* DNA modification detection.

Epigenomic sequencing technologies have enabled, for example and among others, non-invasive epigenomic molecular phenotyping of the human brain (127), rapid epigenomic diagnosis of brain cancers (128), and inference of epigenomic cell-state dynamics (129).

#### Epitranscriptomics

Epitranscriptomics seeks to elucidate the role of RNA structure and modifications in regulating gene expression, where RNA modification focuses on modified nucleotides in mRNA (130).

Sequencing-based methodologies for mapping RNA structures can be classified into enzyme-based *in vitro* methods, chemical-based *in vitro* methods, and chemical-based *in vivo* methods (25). Enzyme-based *in vitro* approaches include parallel analysis of RNA structure (PARS) (131), fragmentation sequencing (FragSeq) (132), parallel analysis of RNA structures with temperature elevation (PARTE) (28), and protein interaction profile sequencing (PIP-seq) (133). These methods are limited to *in vitro* structural analysis and leverage different ribonucleases (RNases; including RNase V1, RNase S1, RNase P1, and RNase T1) to generate a mixture of RNA fragments that, once been analyzed by sequencing, allows for RNA secondary structure inference. Chemical-based methods utilize small membrane permeable molecules such as nucleobase-specific chemicals, carbodiimide modifying reagents, and ribose-specific probes to interrogate RNA structure, which can be applied both *in vitro* and *in vivo* and often capable of achieving single-nucleotide resolution. Chemical-based *in vitro* methods include structure-seq (134), dimethyl sulfate sequencing (DMS-seq) (135), and high-throughput sequencing for chemical probing of RNA structure (Mod-seq) (136). Chemical-based *in vivo* methods include chemical inference of RNA structures sequencing (CIRS-seq) (137), selective 20-hydroxyl acylation analyzed by primer extension and mutational profiling (SHAPE-MaP) (138), *in vivo* click selective 2-hydroxyl acylation and profiling experiment (icSHAPE) (139), and mapping RNA-RNA interactome and RNA structure *in vivo* (MARIO) (140). Combining data from both chemical- and enzyme- based sequencing approaches is considered ideal to gain a more comprehensive RNA structural information.

While chemical modifications on RNA are evolutionarily conserved traits of structural RNAs such as rRNA and tRNA (141), their presence in lncRNAs and small regulatory RNAs (srRNAs) has attracted renewed attention to define their roles in gene expression regulation and disease initiation/progression. The rapid evolution of RNA sequencing technologies has enabled the development of methodologies to interrogate the topography of RNA modifications in the whole transcriptome.

The 13 chemical modifications identified so far in mRNAs can be divided into two categories, i.e., modifications of nucleotides adjacent to the 5' cap and internal RNA modifications. Cap-adjacent nucleotide modifications typically regulate RNA stability and translation, and can occur in mRNA, primary miRNA transcript, lncRNA, small nucleolar RNA (snoRNA), and small nuclear RNA (snRNA) (142). Internal modifications can reside in 5' and 3' untranslated regions (UTRs), coding regions, and mRNA introns, with m6A (N6-methyladenosine) and adenosine to inosine (A to I) editing being the most abundant (143). Internal modifications participate in a diverse spectrum of gene regulatory programs such as mRNA splicing, 3'-end processing, export, stability, localization and translation (144).

As the abundance of mRNA modifications is in general low, large amounts of mRNAs are needed or very deep sequencing is required using current experimental approaches. This has catalyzed the generation of many sequencing-based approaches for transcriptome-wide mapping of mRNA modifications. The most prevalent approach is RNA immunoprecipitation (RIP) sequencing (RIP-seq), where modified nucleotides are recognized by antibodies followed by whole transcriptome sequencing (RIP-seq) (145). Other approaches such as PA-m6A-seq (146), miCLIP-seq (147), m1A-MAP (148), and m7G-MeRIP-seq (149) have been established to enhance the resolution to a single base level taking advantages of nucleotide mismatches or truncation signatures induced to the modified nucleotides before reverse transcription and sequencing by crosslinking antibodies. LAIC-seq can differentiate m6A methylation levels between mRNA isoforms without prior fragmentation at the expense of losing the positional information (150).

Besides, using chemical reactions specific to a given RNA modification followed by short read sequencing provides an alternative approach for RNA modification detection. For instance, RNA bisulfite sequencing (RNA-BisSeq) is one of these approaches that relies on chemical deamination of cytidine to uridine by sodium bisulfite, leaving m5C intact (151–153). A similar yet different category of approaches is enzyme-based, which includes, e.g., MAZTER-seq (154) and m6A-REF-seq (155). In these approaches, the endoRNases MazF and ChpBK cut unmethylated RNA at ACA and UAC motifs without touching m6A methylated RNA (154, 155).

Epitranscriptomic sequencing technologies have enabled us to profile the landscape of epitranscriptomic RNA modifications (156) toward enhanced understandings of biological systems such as a prototype baculovirus (157).

#### DNA-RNA Interactomics

Given the prevalence of DNA-RNA interactions at the transcription start sites (158, 159), diversified modes of chromatin-RNA interactions (160–162), and the high correlation between RNA-chromatin attachment and histone modification events such as H3K27ac and H3K4me3 (158), a variety of tools have been established to investigate the DNA-RNA interactome. These technologies can be divided into two categories, i.e., mapping genome-wide locations of a specific RNA, and mapping all chromatin-interacting RNAs together with their genomic interacting regions.

Methods fell into the first category include chromatin isolation by RNA purification (ChIRP) (163), capture hybridization analysis of RNA targets (CHART) (164), and RNA antisense purification (RAP) (160). These approaches take advantages of biotinylated complementary oligonucleotides to pull down a specific target RNA together with its binding partners followed by characterization of these binding molecules through sequencing or MS.

Technologies of the second category include mapping RNA–genome interactions (MARGI) (158), chromatin-associated RNA sequencing (ChAR-seq) (165), and mapping global RNA interactions with DNA by deep sequencing (GRID-seq) (159). These methodologies leverage crosslinking reagents to preserve DNA-RNA interactions followed by proximity ligation to convert RNA and its binding DNA into a chimeric sequence complex before sequencing.

Approaches of this category have been used for, among others, identifying *de novo* targets of chromatin-bound RNAs including nascent transcripts, chromosome-specific dosage compensation ncRNAs, and genome-wide trans-associated RNAs participating in co-transcriptional RNA processing (165, 166).

#### RNA-RNA Interactomics

The diversity, flexibility and complexity of the RNA kingdom regarding the types and molecular functions of RNAs have rendered RNA-RNA interactomics a unique omics layer that had attracted much attention.

Methodologies developed for probing RNA-RNA interaction had once been restricted by the prior knowledge on one of the interacting RNAs such as X-ray crystallography, nuclear magnetic resonance (NMR), and psoralen cross-linking (167). The discovery of chimeric RNAs from transcriptome data has made it possible to detect *de novo* RNA-RNA interactions despite the low prevalence of chimeric RNAs (168). Identifying RNA-RNA interactions by purifying proteins bringing RNA interactants together is the rational of RNA hybrid and individual-nucleotide resolution UV crosslinking and immunoprecipitation (hiCLIP) (169) and crosslinking, ligation, and sequencing of hybrids (CLASH) (170). Yet, both approaches differ in the utility of antibody-based isolation of target protein in hiCLIP and using ectopic expression of a tagged protein in CLASH.

Methods established for high-throughput RNA interactome analysis leverage proximity ligation to produce chimeric sequences, which include, e.g., psoralen analysis of RNA interactions and structures (PARIS) (171), sequencing of psoralen-crosslinked, ligated, and selected hybrids (SPLASH) (172), ligation of interacting RNA followed by high-throughput sequencing (LIGR-seq) (173), and MARIO (140). These methods share similar protocols that contain *in vivo* RNA crosslinking, RNA fragmentation, intramolecular ligation and reverse crosslinking. It is noteworthy that the choice of crosslinking reagents may determine the types of RNA interactions to be discovered. For example, psoralen or its derivatives as used in PARIS, SPLASH, and LIGR-seq intercalate in RNA helices and can be used to identify hybridized RNA pairs.

Interrogating the RNA-RNA interactome has enabled us to construct the higher-order transcriptome structure of living cells that guided the discovery of lncRNA structures and functionalities (171), has aided in defining the principles of how RNAs interact with themselves and with other RNAs in gene regulation and ribosome biogenesis (172), and has helped in revealing novel interactions between snoRNAs and mRNAs (173).

#### DNA-Protein Interactomics

Interactions between proteins and DNA play fundamental roles in transducing genetic information into functionalities. Methods for characterizing such interactions include electrophoretic mobility shift assays, DNase footprinting, ChIP, and systematic evolution of ligands by exponential enrichment (SELEX) (174–177) which, however, are only useful if the DNA remains intact.

Protein binding microarrays have also been adopted to investigate transcription factor (TF)-DNA interactions; yet, the binding sites of many TFs are longer than those that can be combined to the array (178). ChIP-hybridized association mapping platforms provide another category of approaches to investigate protein-DNA interactions (179). SELEX, coupled with NGS, represents a high-throughput solution for studying protein-DNA interactome which, however, is not feasible to use if DNA is cleaved during protein binding (180). These aforementioned platforms unanimously require the known identify of the protein of interest, with approaches capable of identifying *de novo* protein-DNA interactions urgently called for.

ChIP-Chip, also known as the genome-wide location analysis, combines chromatin immunoprecipitation (ChIP) and DNA microarray analysis to identify protein-DNA interactions occurring in living cells (181). It has been considered as the conventional approach used in analyzing histone modifications before the invention of NGS, which shares the same drawbacks as ChIP (e.g., constrained by the availability of an organism-specific microarray). ChIP sequencing, coupling ChIP with NGS, outweighs ChIP-Chip by generating outputs with higher spatial resolution, dynamic range, and genomic coverage and, importantly, capable of exploring any species with a sequenced genome in principle (181, 182). In ChIP sequencing, DNA-bound proteins are immunoprecipitated by specific antibody followed by extraction, purification, and sequencing of the bound DNA. This enabled us to interrogate histone modifications and the interactome that offer deep insights into genomic regulatory events.

#### RNA-Protein Interactomics

The pervasive transcription of the genome creates the diversified reservoir of ncRNAs. Despite our limited knowledge regarding the functions and regulatory mechanisms of ncRNAs, it has been well-accepted that interactions between RNA binding proteins (RBPs) and RNAs play vital roles in cell homeostasis maintenance. The dynamic combination, competition and coordination between RBPs and RNA offer many pointcuts to the mechanisms of action of these RNAs. Many experimental techniques have been established to systematically investigate such interactions. High-throughput sequencing of RNA isolated by cross-linking immunoprecipitation, namely HITS-CLIP or CLIP-seq (39), as well as its variants such as photoactivatable-ribonucleoside-enhanced cross-linking and immunoprecipitation (PAR-CLIP) (183), individual-nucleotide resolution cross-linking and immunoprecipitation (iCLIP) (184), cross-linking and analysis of cDNAs (CRAC) (185) and cross-linking, ligation, and sequencing of hybrids (CLASH) (167) have been consecutively established to decode protein-RNA interactomes.

While these technologies have been successfully applied to decode miRNA-target interactions (186), identify RNA binding sites of splicing factors (187, 188), investigate epigenetic modification-associated RNAs (189), and explore functions of ceRNAs (190), they suffer from several limitations that await additional improvements. These technical issues include, e.g., how to further reduce the background noise [that though is already lower than RIP (191)] and simultaneously enhance RNA output efficiency to study low abundance RBPs, as CLIP achieves a high signal-to-noise ratio at the cost of RNA abundance (40).

### MS-Based Omics

#### Proteomics

Proteomics investigates the functional relevance of all expressed proteins in a cell, tissue or organism [namely proteome (192)] by interrogating the information flow through protein signaling (193). Since most biological functionalities are actioned by proteins, it is important to reliably measure proteome alterations during cellular state transitions such as in the context of carcinogenesis (2).

MS is a primary protein characterization technique that can be used to determine the amino acid sequence of a protein and PTM sites. MS measures charged molecules based on their mass-to-charge ratios, where the mass analyzer characterizes molecules by their mass-to-charge ratios, and the signal intensities of charged peptides reflect the quantity. One significant advance brought by MS to the omics field is the rate at which it identifies proteins in an entirely discovery-driven way. The advent of the high-resolution “LTQ™Orbitrap™ (194)” MS instruments coupled with powerful analytical tools such as MaxQuant enabled the first draft of human proteome (194).

There are several types of commercially available mass spectrometers. These include high-resolution MS methods such as LTQ™Orbitrap™ (194), matrix-assisted laser desorption ionization-time of flight-time of flight (MALDI-TOF-TOF), and Fourier transform ion cyclotron resonance (FT-ICR) that measures the exact masses [i.e., the theoretical mass of specific isotopic composition of a charged molecule (195)], and low resolution MS approaches such as quadrupole and ion-trap that measure the nominal mass [defined as the integer mass of the most abundant stable isotope of a molecular ion (195)].

Various tandem mass spectrometric techniques (MS/MS) approaches have been established to generate unique fragmentation profiles from different proteins that enables *de novo* protein characterization and isobaric peptides differentiation (196). In MS/MS, peptides isolated from the first mass analyzer are subjected to the second mass analyzer on collision with a neutral gas or interaction with activated electrons, where sequential fragmentation can be performed as needed. Several MS/MS platforms are available including electron-based approaches such as electron capture dissociation (ECD) (197), electron-induced dissociation (EID) (198), electron transfer dissociation (ETD) (199), and collision-induced dissociation (CID) where fragment ions are generated from collision with neutral gases (200). It is often to collect the structural information of the target molecules using different MS/MS methods that are complementary in revealing the true identities of unknown proteins (201).

Liquid chromatography-mass spectrometry (LC-MS) and gas chromatography-mass spectrometry (GC-MS) are MS coupled with chromatography-based separation modules that convey several advantages over MS. One critical benefit of using LC or GC before MS is the alleviation of the matrix effect and ion suppression by separating analytes from interfering endogenous compounds such as salts and ion pairing agents using an appropriate chromatography. Also, peptides can be quantified by measuring the area under the chromatographic peak. LC-MS has been used to characterize various kinds of proteins with broad ranges of physicochemical properties and molecular weights, and the use of GC-MS in protein characterization is rather rare that has been limited to profile relatively more volatile molecules with lower molecular weights such as protein adducts.

Proteome interrogation has diverse applications such as identifying novel ceramide-binding proteins (202), analyzing *Ophiocordyceps sinensis* at different culture periods (203), and interrogating the proteomic landscape of cardiometabolic diseases (204).

#### Metabolomics

Metabolites, typically defined as low molecular weight biomolecules (<1,500 Da) participating in cell endogenous metabolism, function as energy sources, signaling molecules, and metabolic intermediates with protein modulatory roles (see section on epiproteomics) in complex biological systems. Metabolites have been demonstrated to serve as important biological modulators across multilayer omics toward the maintenance of cellular homeostasis. Metabolome is referred to as a collection of all metabolites in a cell that encompass all biomolecules except for the genome, transcriptome, proteome, and metals.

Methods for metabolome interrogation include Fourier transform-infrared (FT-IR) spectroscopy (205), Raman spectroscopy (206), NMR spectroscopy (18, 207), MS-based approaches such as MS (20), MS/MS (208), liquid chromatography (LC)-MS (209), gas chromatography (GC)-MS (210, 211), as well as others.

FT-IR, Raman and NMR spectroscopies provide non-destructive and rapid solutions for metabolite analysis, where absorption spectra at specific wavelengths determines the structure of unknown metabolites and the area under the curve (AUC) of the absorption spectra quantifies the amount. However, these approaches do not have sufficient sensitivity and selectivity (212–215).

MS is the most feasible tool to probe the metabolome that can detect a wide spectrum of metabolites (216). Isobaric metabolites can be distinguished by various MS/MS (208) and/or being coupled with LC (209) or GC (210, 211).

Metabolomics is comprised of targeted and non-targeted metabolomics. While targeted metabolomics addresses specific biological hypotheses by providing quantitative information for target metabolites involved in specific metabolic pathways, non-targeted metabolomics generates hypotheses and can be used for *de novo* target identification by charactering as many metabolites as possible in a biological sample (217).

Metabolome profiling has been used in identifying candidate genes and metabolites (218), as well as revealing the metabolic mechanism of the therapeutic efficacy (219).

#### Epitranscriptomics

Besides sequencing-based detection approaches, RNA modification can also be investigated using LC-MS/MS. In these approaches, total RNA or purified mRNA are digested into individual nucleotides followed by LC-MS/MS, and the presence and quantification of all RNA modifications can be determined by comparing the MS peaks from the sample with that of standards (30, 149, 220). Though these methods are quantitative and the results are concordant across studies, they require large amounts of input samples and are not feasible for detecting low abundance nucleotides such as caps, and do not provide information on the location of the modified positions.

Epitranscriptome landscape has been used to decode the atlas of RNA modifications (221), and characterize the topology of human and mouse m5C epitranscriptome (222).

#### Epiproteomics

Epiproteome includes PTMs occurring in histone and non-histone proteins such as protein phosphorylome, methylome, acetylome, ubiquitinome, SUMOylation, and newly discovered lactylome, succinome, and etc. While histone constitutes as an important player in the protein-DNA interactome and takes a central role in shaping the epigenome (223), and non-histone proteins participate in signal relay and actualize genetic information into cell functions, epiproteome of various kinds offer a unique view on how epigenetic regulations impact critical events associated with the central dogma and cell behavior through marking proteins with varied epiproteomic barcodes.

Microsequencing is the first approach ever used for epiproteomic studies that adopts Edman degradation to determine protein sequence, which is time-consuming and requires a large amount of highly purified sample (31). Later, antibody-based methods such as western blotting, immunofluorescence analysis and ChIP gained a wide popularity in low-throughput PTM studies. However, antibody-based assays rely on modification-specific antibodies which are not always available, require a priori knowledge of the type and position of the modification of interest, and are not capable of measuring multiple PTMs occurring within the same protein. MS-based proteomics has enabled the characterization of protein PTMs in a high-throughput manner. MS-based methodologies enable unbiased profiling of diverse modifications simultaneously, quantitative analysis of protein modifications, and *de novo* identification of unknown modification patterns.

MS-based strategies for epiproteome investigation can be classified into three categories, i.e., “bottom-up” where a target protein is proteolytically digested into short peptides (5–20 Aa) prior to MS analysis, “top-down” that defines the proteomes present in a sample through analyzing intact proteins, and “middle-down” that is designed for analyzing histone PTMs as histone N-terminal tails can be cleaved off by specific proteases to generate polypeptides with accessible size for MS detection. The “top-down” approach in a high-throughput fashion can depict a comprehensive and accurate view on the epiproteome of the targeted system, but is challenging as larger molecules are difficult to be separated by LC and analyzed by MS, and the chance of having isobaric proteomes (species sharing the same mass and similar physico-chemical properties such as H3K27me1K36me2 and H3K27me2K36me1) increases with the portion of protein analyzed (224).

Phosphoproteome is perhaps the most pervasive PTM landscape, the analysis of which adopts the “bottom-up” approach. Peptide digests are typically enriched (through the use of, e.g., immunoaffinity chromatography, ion exchange chromatography, immobilized metal ion affinity chromatography, metal oxide affinity chromatography, chemical derivatization), fractioned off- or on-line (by chromatographic separation techniques such as reversed phase high performance liquid chromatography) prior to mass spectrometry analysis (225). A recently commercialized aerodynamic high-field asymmetric waveform ion mobility spectrometry (FAIMS) device was recently incorporated into the phosphoproteomic workflow to aid in the gas-phase fractionation, which resulted in the identification of around 15–20% additional phosphorylation sites and a 26% increase of the reproducibility (226).

Epiproteome interrogation has been largely applied to capture the dynamic phosphoproteome profile of a particular biological system such as prostate cancer (227), human urine (228), influenza A/B (229), and budding yeast (230), or map cellular alterations in response to specific perturbations (231) such as HIV-infected brain (232).

#### Protein-Protein Interactomics

Protein-protein interactions (PPIs) represent the most prominent and well-studied molecular interactions within cells. The earliest high-throughput technology employed for PPI identification is yeast two hybrid (Y2H) screening, which works by separating two functional domains of a single TF that elicits signals once brought into close proximity (233). Y2H screening is laboriousness and cannot identify multi-protein complexes in one run (42). MS-based protein characterization allows rapid identification of multiple interacting proteins without the need of antibodies (234–236), with the outputs easily confirmable using targeted approaches such as IP or immunofluorescence (237). As one example, ~5,700 proteins and over 27000 complex PPIs were identified using LC-MS/MS and merged into a protein-protein interactome, termed BraInMap (238). Lastly, it is worth to mention co-immunoprecipitation (coIP-MS) followed by mass spectrometry, which is a common practice to interrogate proteins interacting with a given protein bait (44).

A high-throughput screening approach has been established to characterize PPIs directly from cell-free protein synthesis reactions. Proteins of interest are immobilized non-covalently on the donor and acceptor beads that produce chemiluminescent signals on interactions. This technique relies on the Amplified Luminescent Proximity Homogeneous Linked Immunosorbent Assay (AlphaLISA) that enables rapid PPI characterization without the need for protein purification and a highly parallel and miniaturized workflow taking advantages of robotic and acoustic liquid handling. This recent technology can characterize competitive binding of proteins for specific epitopes besides direct PPIs, and thus has been applied to screen candidate antibodies capable of competing with the spike receptor-binding domain (RBD) of SARS-CoV-2 for human angiotensin-converting enzyme 2 (ACE2) (239).

#### Protein-Metabolite Interactomics

Protein-metabolite interactions are essential in maintaining cell homeostasis under the conventional state, and coordinating responses to internal or external stress or perturbations. Protein-metabolite interactions are prevalent in cells which were estimated to be at the scale of millions (240–242).

Early methods exploring protein-metabolite interactions adopt protein tagging or metabolite modification (46, 243) and are limited to the detection of protein-lipid interactions and those involving hydrophobic molecules (244–246). A systematic and unbiased method to identify *de novo* protein-metabolite interactions was proposed, under the hypothesis that the binding of a metabolite to a protein of interest can block its Proteinase K cleavage sites (247). In this approach, proteins are extracted under non-denaturing conditions with metabolites being cleared off using size-exclusion chromatography; a specific metabolite is added to an aliquot of the protein followed by Proteinase K-mediated proteolysis of both metabolite-containing and metabolite-free proteomes and trypsin-mediated complete digestion; the MS output of metabolite-bound proteome is expected to contain two non-tryptic termini whereas the other one does not (247). Though this approach offers a non-biased solution without the need of any chemical modifications, it does not provide a comprehensive set of protein-metabolite interactions as currently available MS cannot detect all peptides within any enzymatically digested samples. Another critical issue of this approach that challenges its application in eukaryote cells is the loss of compartmentation information by cell lysing (247). PROtein-Metabolite Interactions using Size separation (PROMIS), a simultaneous global interrogation tool of the protein-metabolite interactome, has been developed that is featured by low false positives related to a high concentration of the bait molecule (proteins or metabolites) and low false negatives related to small-molecule modifications (47). An NMR-based approach permitting the direct detection of interactions between any set of water-soluble proteins and metabolites was proposed (48), and applied to investigate protein-metabolite interactions in the central metabolism of *Escherichia coli* (248). An approach based on high-resolution NMR relaxometry that does not require any invasive procedure or separation step was established to detect weak metabolite-macromolecule interactions in complex media such as biological fluids (49).

Through interrogating the protein-metabolite interactome, novel enzyme-substrate relationships and cases of metabolite-induced protein complex remodeling have been identified (247), and a wide range of proteins participating in lipid pathways have been pharmacologically characterized in mammalian cells, among which a selective ligand for NUCB1 (a compound that perturbs the hydrolytic and oxidative metabolism of endocannabinoids in cells) was identified (244).

## Knowledge-Based Omics

The concept of “omics” has been extended from a set of experimental and computational approaches as well as a particular layer of molecular information interrogated using these established tools to a cocktail of knowledge gained by integrating multiple omics data in a particular research domain. This concept shift has led to the generation of omics such as immunomics and microbiomics (Figure 1, Table 1).

### Immunomics

The term of “immunomics” was firstly introduced in 2001 (249), and refers to the interrogation of immunology through the integration of information from genomics, proteomics and transcriptomics, with the aim of translating molecular immunology into clinics (250). The immunome can be defined as the set of antigens or epitopes that interface with the host immune system (251). Immunomics has been used to improve disease diagnosis, treatment and prevention (252), with the potential of revolutionizing our rational on vaccine design and antigen discovery (250).

### Microbiomics

Microbiomics is the science of collecting, characterizing and quantifying molecules responsible for the structure, function, and dynamics of a microbial community by integrating multiple omics information such as genomics, transcriptomics, proteomics and metabolomics, where all microorganisms of a given environment, called microbiome, are analyzed to study the potential role that such microorganisms have in diseases (253, 254). Human microbiome, emerged in 2008 from the human microbiome project (http://commonfund.nih.gov/hmp/index), is comprised of trillions of microbes inhabiting inside the human body and interacting with their host (255). Evidence from rodent models of microbiome studies has suggested strong associations between microbiomes and human diseases (256), and microbiome can provide unique insights into human diseases, with unprecedented ability demonstrated in interrogating and modulating the communities that co-inhabit with human (257, 258).

## Trends in Omics Technology Development

The suffix “omics” represents the revolutionary technological advancement made in the past three decades that enables us to simultaneously analyze thousands of molecules. Genomics, transcriptomics, proteomics and metabolomics, namely the “four big omics” (240), have led to the creation of their epiomics (epigenomics, epitranscriptomics, and epiproteomics) and interactomics (e.g., DNA-RNA interactomics, RNA-RNA interactomics, DNA-protein interactomics, RNA-protein interactomics, protein-protein interactomics, and protein-metabolite interactomics) which are technology-based, and other omics such as immunomics and microbiomics that are knowledge-based.

**Figure 2:**
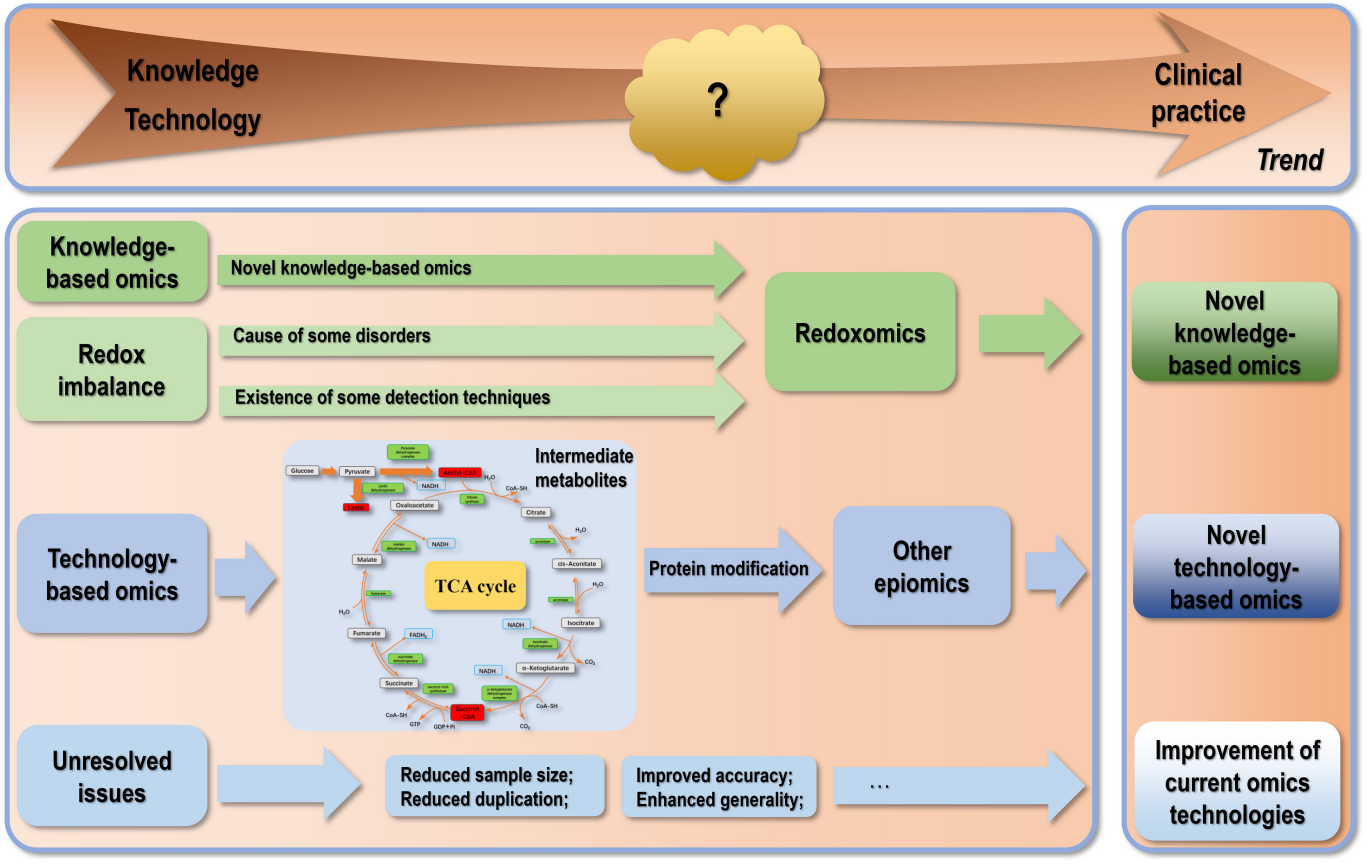
Conceptual illustration on the future trend in omics technology development. There are three trends regarding the developmental paths of omics technologies. The first category of tasks is to resolve the technical problems existing in current omics techniques. The second trend is the identification of novel types of omics especially novel epiomics derived from modifications by various intermediate metabolites. With increased understanding on the importance of cell homeostasis at various levels regarding human disease management, there is a trend of cutting into a particular knowledge domain from a systematic angle via omics data integration as demonstrated by immunomics and microbiomics. In this trend, we propose “redoxomics” as an emerging type of knowledge-based omics given the critical roles of redox homeostasis in maintaining cells at the healthy state and the pathogenesis of various diseases including cancers.

Despite the abundancy and diversity of the experimental and computational approaches available for omics interrogation, translating knowledge gained by diving into varied levels of omics into clinical practice is still at its nascent stage. Whether omics studies can significantly impact disease diagnosis and therapeutics depends on the resolvent of issues from the technical aspect and existing in the knowledge extraction process. Technical problems include, e.g., how to substantially reduce the sample amount without sacrificing statistical accuracy when the sample is rare and precious, how to reduce replicates without negatively affecting output stability, and how to increase the accuracy of the output by reducing both false negative (FN) and false positive (FP) rates. The reproducibility of MS-based omics is, in general, lower than sequencing-based omics. For example, the results of some MS-based omics such as proteomics, metabolomics and their epiomics are highly dependent on the type of mass spectrometer, as well as the sample processing protocol and data processing pipeline used. This renders the compromise of the accuracy and reproducibility of some aforementioned omics unavoidable in pursuit of the global landscape. Domain specific languages such as Nextflow (259) have been established to help partially resolve the reproducibility issue by sharing the codes and workflows used for data analysis. Different omics interrogation approaches have different FN and FR rates, depending on many computational indexes such as the calling pipeline parameters and read coverage. Take short-read NGS as an example for sequencing-based omics, the FN rate was reported to vary between ~6 and 18%, and the FR rate was <3% (260). The FN and FR rates for MS-based omics are, in general, higher than that of sequencing-based omics due to inadequate capture of some protein contents in a sample (FN) or misidentification of a chemically modified peptide as a biological variation (FR) (261). Concerns impeding the knowledge gaining process include, e.g., the heterogeneity across studies regarding the study design, sample size and sampling approach, treatment, and follow-up duration, leading to the little generality of the varied outputs consecutively being reported (Figure 2).

As we learn more about known cell components, novel types of biological players are being discovered that further complicate our existing knowledge on how human cells behave under physiological and pathological conditions. Besides, the number and variety of cells comprising the system to be studied regarding human health has been expanded by an order of magnitude by microbiome (262). These technological advances sometimes make our efforts toward complete understanding of human cell system frustrating as the finishing line seems to be at an ultimate distance that can never be reached given the consistently being identified new factors with prominent roles on the cell machinery. For instance, diversified types of protein modifications have been recognized such as acetylation, lactylation, succination, and crotonylation in the past few years, most modifiers of which are intermediates from the ATP production processes such as glycolysis and the TCA cycle. This not only centers the role of metabolism in cell behavior regulation, but also inspires us to think whether other intermediates (such as fumarate) also regulate protein functionalities through creating marks on proteins? Is this one of the primary routes that external intakes (such as food) regulate cellular behavior that bridges the gap between cell metabolism and functionalities? These, by all means, deserve our deep thinking and represent interesting topics to explore (Figure 2).

Another important factor contributing to the huge complexity of cells that challenges translational omics is the multi-layer heterogeneity. Omics technologies at a particular level can only present one picture of a cellular system that dynamically transits among varied states. In addition, cells presented in tissues or biopsies can be very heterogeneous, rendering accurate interpretation of omics information challenging. Further, differential clinical manifestations and treatment responses among patients add an additional layer of complexity regarding the clinical use of omics technologies.

Accordingly, there had been a trend of integrating multiple omics information toward improved understanding of a particular knowledge domain as evidenced by the generation of immunomics and microbiomics. This enables us to portrait a systematic view on a research area that gains additional value if the topic conveys systematic impacts on human cells regarding its decision toward either homeostasis maintenance under the healthy state or running into a chaotic state that drives cells malignant.

Redox imbalance has been indicated as a causal factor of a variety of disorders such as cancer (263–265) and hypertension (266) with translational significance. Besides, reactive oxygen and nitrogen species (RONS) are also important signaling molecules with known roles on normal function regulation such as insulin and growth factor signaling (267). Also along with this line is oxidative cysteine modification that had been considered as a central PTM event associated with many diseases (268–270). The significance of redox homeostasis in disease state control may characterize redox biology at the omics scale, possibly named as “redoxomics,” which offers new avenues for therapeutic intervention (Figure 2). Already there exist some strategies to characterize protein thiol modifications (271), making investigations on redoxomics technically possible. Methods of this kind can be categorized to MS-based modalities that require efficient trapping of the native redox state of the thiol proteome given the labile nature of cysteine residues. In these methods, thiols are protonated by strong acid followed by free thiol blockage using a reactive thiol alkylating reagent such as N-ethyl maleimide (NEM) or iodoacetaminde (IAM); Cysteine residues are then labeled by, e.g., isotopically modified derivatives of thiol alkylating reagents, and characterized by LC-MS, LC-MS/MS or peptide mass fingerprinting (272). In most cases, this is just the first step toward cysteine modification, and orthogonal technology development represents an essential research direction that may lead the trend in omics technique development if redoxomics gains sufficient attention as it deserves.

By leveraging multiplexed fluorescence, DNA, RNA and isotype labeling, and taking advantages of multiomics data interrogation, “spatial omics” has emerged to enable the detection of variations in, e.g., transcriptome and proteome, within their native spatial context (273) and been commonly coupled with single cell technologies (274). Spatially resolved transcriptomics (SRT) is the first and most well-developed technology of this kind that identifies gene expression profiles in tissue biopsies prior to histopathological annotations. Following the trend in multiple omics integration, techniques on spatial multiomics have been established by integrating whole SRT with immunofluorescence protein detection in the same tissue section, allowing the gaining of a holistic view on variations in colocalized protein and gene expression profiles with tissue organization (275). Visium, a software provided by 10 × Genomics, can be used for constructing high-resolution microscopic images with transcriptomic data aligned to the tissue footprint, and extended for interpreting spatial multiomics. The authors anticipate an emerging trend of other “info-omics” beyond “spatial omics” in the future by integrating omics with other dimension of information such as “time” in the form of, e.g., treatment duration, and follow-up time.

Last but not the least is the critical contribution of bioinformatics to omics interrogation. Various toolboxes have been made available for, e.g., data quality control, pre-processing, abnormal molecule identification, interaction prediction, enrichment analysis, pathway analysis, network construction, as well as more advanced or focused analyses such as pseudo-time and trajectory inference in single cell sequencing. Besides, numerous databases and computational tools have been developed to allow for the easy access and analysis of omics data. The Cancer Genome Atlas (TCGA) (276) and Gene Expression Omnibus (GEO) (277), among others, have been frequently used for omics data deposit and retrieval. Genome Sequence Archive (GSA) (278) developed by the Chinese National Genomic Data Center has been launched to compliment this giant-size database portfolio, with GSA-human recently announced as part of GSA to provide a repository for human genetic related omics data (279). Various databases smaller in size and/or with more specialized focuses have been established by different groups such as “LCMD” for lung cancer metabolome (280), “DBSAV” for deleterious single amino acid variation prediction in human proteome (281), “SARS-CoV-2 3D” for coronavirus proteome (282), and “CMVdb” for cytomegalovirus multi-omes (283). A plethora of machine-learning algorithms and computational tools have been developed to interrogate these omics data toward gained knowledge or new discoveries such as WeiBI for PPI taking into account of functional enrichment (284), DTI-MLCD for drug-target interactions utilizing multi-label learning (285), and MDF-SA-DDI for predicting interaction events between two drugs based on a transformer self-attention mechanism (286). We foresee a reciprocal transformation between our dry and wet lab powers in omics investigation, i.e., while technical advances urge the development of novel bioinformatics tools, intelligent computational strategies broaden our horizon on the complexity of the biological network that urges the emergence of novel experimental approaches. This will boost both fields flourish toward our enhanced abilities for cellular omics interrogation.

## Author Contributions

XD conceptualized the idea, drafted the paper, provided financial support, and final proved the manuscript. XD and LS conducted literature searching and prepared figures and tables. All authors agree with the content and its publication.

## Funding

This study was funded by the National Natural Science Foundation of China (Grant No. 81972789) and Fundamental Research Funds for the Central Universities (Grant No. JUSRP22011).

## Conflict of Interest

The authors declare that the research was conducted in the absence of any commercial or financial relationships that could be construed as a potential conflict of interest.

## Publisher's Note

All claims expressed in this article are solely those of the authors and do not necessarily represent those of their affiliated organizations, or those of the publisher, the editors and the reviewers. Any product that may be evaluated in this article, or claim that may be made by its manufacturer, is not guaranteed or endorsed by the publisher.
